# Unusual phenotypes in patients with a pathogenic germline variant in *DICER1*

**DOI:** 10.1007/s10689-021-00271-z

**Published:** 2021-07-31

**Authors:** Kateryna Venger, Miriam Elbracht, Julia Carlens, Peter Deutz, Felix Zeppernick, Lisa Lassay, Christian Kratz, Martin Zenker, Jung Kim, Douglas R. Stewart, Ilse Wieland, Kris Ann P. Schultz, Nicolaus Schwerk, Ingo Kurth, Udo Kontny

**Affiliations:** 1 Division of Pediatric Hematology, Oncology and Stem Cell Transplantation, Department of Pediatrics, Medical Faculty, RWTH Aachen University, Aachen, Germany; 2 Institute of Human Genetics, Medical Faculty, RWTH Aachen University, Aachen, Germany; 3 Clinic for Pediatric Pulmonology, Allergology and Neonatology, Hannover Medical School, Hannover, Germany; 4 Department of Gynecology and Obstetrics, Medical Faculty, RWTH Aachen University, Aachen, Germany; 5 Department of Obstetrics and Gynecology, University Hospital Giessen, Giessen, Germany; 6 Clinic for Pediatric Hematology and Oncology, Hannover Medical School, Hannover, Germany; 7 Institute of Human Genetics, University Hospital Magdeburg, Magdeburg, Germany; 8 Division of Cancer Epidemiology and Genetics, National Cancer Institute, NIH, Rockville, MD USA; 9 International PPB/DICER1 Registry, Minneapolis, MN USA; 10 Cancer and Blood Disorders, Children’s Minnesota, Minneapolis, MN USA

**Keywords:** *DICER1*, Unusual phenotype, Developmental delay, Skeletal findings, Pierre-Robin sequence

## Abstract

Pathogenic germline *DICER1* variants are associated with pleuropulmonary blastoma, multinodular goiter, embryonal rhabdomyosarcoma and other tumour types, while mosaic missense *DICER1* variants in the RNase IIIb domain are linked to cause GLOW (global developmental delay, lung cysts, overgrowth, and Wilms’ tumor) syndrome. Here, we report four families with germline *DICER1* pathogenic variants in which one member in each family had a more complex phenotype, including skeletal findings, facial dysmorphism and developmental abnormalities. The developmental features occur with a variable expressivity and incomplete penetrance as also described for the neoplastic and dysplastic lesions associated with *DICER1* variants. Whole exome sequencing (WES) was performed on all four cases and revealed no further pathogenic or likely pathogenic dominant, homozygous or compound heterozygous variants in three of them. Notably, a frameshift variant in *ARID1B* was detected in one patient explaining part of her phenotype. This series of patients shows that pathogenic *DICER1* variants may be associated with a broader phenotypic spectrum than initially assumed, including predisposition to different tumours, skeletal findings, dysmorphism and developmental abnormalities, but genetic work up in syndromic patients should be comprehensive in order not to miss additional underlying /modifying causes.

## Introduction

DICER1 is a member of ribonuclease III (RNaseIII) family and responsible for microRNA processing. Thus, DICER1 modulates gene expression. Germline pathogenic variants in *DICER1* cause a tumour predisposition syndrome (OMIM 601200), which is characterized by occurrence of pleuropulmonary blastoma, Sertoli-Leydig cell tumour, cystic nephroma, multinodular goiter, embryonic rhabdomyosarcoma of the cervix uteri and other tumour types [1].

Mosaic somatic missense *DICER1* variants in the RNase IIIb domain are linked to GLOW syndrome, an acronym from the reported core features of global developmental delay, lung cysts, overgrowth, and Wilms’ tumour (OMIM 618272) [2]. The discussion of whether GLOW syndrome is a separate entity is ongoing: meanwhile some patients with pathogenic germline variants in *DICER1* are reported matching the phenotype of GLOW syndrome and not all mosaic variants in the RNAase IIIb domain lead to the typical GLOW syndrome phenotype but instead are in line with the variable expressivity and reduced penetrance of *DICER1*-associated features [3].

Here, we report four families with germline *DICER1* pathogenic variants. One member in each family had in addition unusual symptoms which could be a hint to a broader phenotypic spectrum.

## Patients

### Case 1

**Fig. 1:**
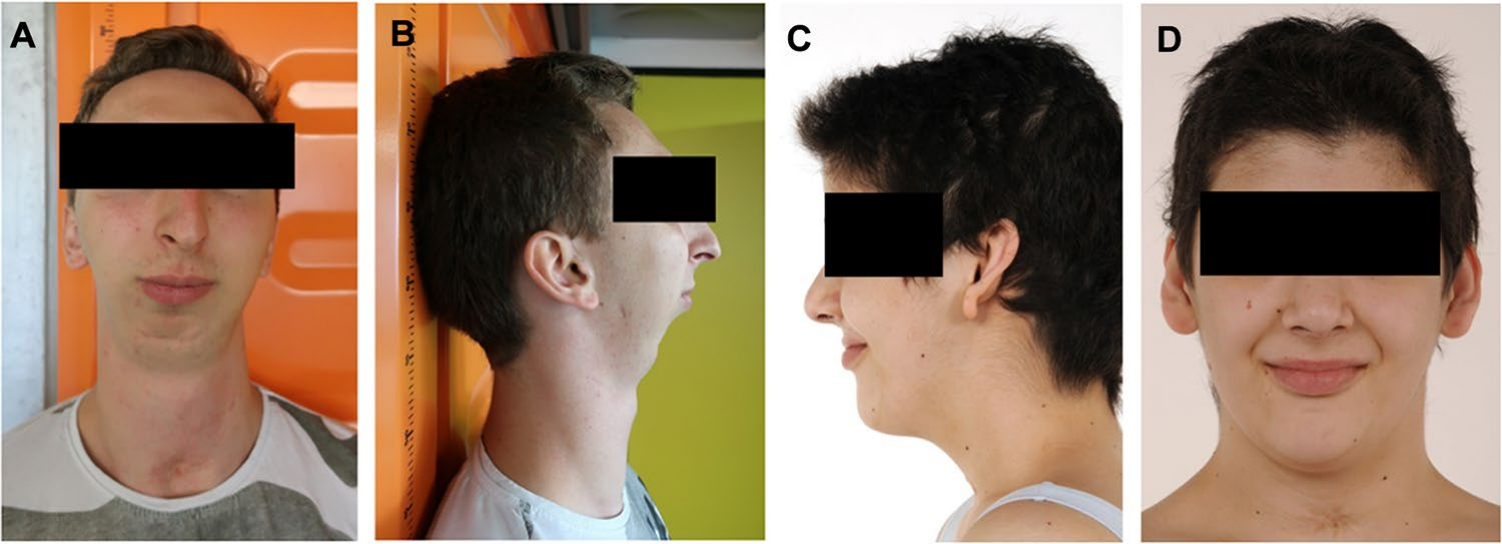
**A**, **B** Front and side view of patient 1 with severe Pierre-Robin-sequence. The swallowing of solid food is hardly possible. **C**, **D** Patient 4 with *DICER1* variant and mild facial features,

The first family includes three siblings and their mother. The oldest son was born with severe Pierre-Robin-sequence (Fig. 1A, B), shortening of the left arm and leg and bilateral hip dysplasia. He later developed multiple thyroid nodules. His sister developed ovarian bilateral Sertoli-Leydig cell tumours at age 5. At age 17, she was diagnosed with papillary thyroid carcinoma. The younger brother also developed papillary thyroid carcinoma at age 17. The children’s mother developed Sertoli-Leydig cell tumours of both ovaries at age 23. Previously, she had a benign thyroid nodule removed at age 12. Later, she developed a follicular thyroid carcinoma, which was successfully treated with radioiodine therapy. The children’s maternal grandmother had developed a renal cell carcinoma, a bone malignancy and thyroid disease, which makes *DICER1* pathogenic variation probable. However, testing in her has not been performed.

### Case 2

This female patient was born as the third child to non-consanguineous parents at 39 weeks of gestation. She showed macrosomia, macrocephaly, and dysmorphic facial features such as a prominent forehead, low set ears, hypertelorism and ptosis. At the age of 3 months she underwent VP-shunt placement for treatment of obstructive hydrocephalus, and Chiari malformation type 1 was noted. At the age of 11 months the patient was diagnosed with Wilms tumour on the left kidney, which was treated with chemotherapy and nephrectomy according to the SIOP 2001 protocol. Large bilateral lung cysts were diagnosed during staging. Lung biopsy showed focal peripheral alveolar cyst formation consistent with congenital cystic adenomatoid malformation type IV. The patient is now 6 years old and shows developmental delay, dystrophy and mild respiratory insufficiency with overall good quality of life. Her family history was unremarkable for DICER1-associated tumors or unusual phenotypic features.

### Case 3

The third individual was found to have gynandroblastoma at age 16 with features of intermediately differentiated Sertoli-Leydig cell tumour with juvenile granulosa cell tumour components with atypia and features of sclerosing stromal tumour. The tumour was completely resected (FIGO Stage IA) and followed with observation. She also has multinodular goiter, macrocephaly, macroglossia, developmental delay, mild bilateral varus forefoot and multiple atypical nevi. Family history is significant for a sibling with gynandroblastoma and multinodular goiter [4]. Multiple family members have a history of talipes equinovarus. Three years after her ovarian tumour diagnosis, she developed a pituitary microadenoma which later resolved. Chest imaging has shown no lung cysts, however, histoplasmosis was incidentally discovered and successfully treated. She remains alive and well 14 years following resection of her ovarian tumour.

### Case 4

The fourth individual was born preterm at 33 weeks of gestation to non-consanguineous parents. She showed mild dysmorphic facial features such as a bulging underlip, hypertelorism, flat nasal bridge, macroglossia, high palate and protruding ears (Fig. 1C, D). Tracheomalacia was noted at birth, requiring a tracheostomy for her first 3 years of life. Later, she showed developmental delay, especially in the field of speech. At age 16 she was diagnosed with an embryonic rhabdomyosarcoma of the cervix uteri. Further work-up revealed multiple thyroid nodules, atrophy of both optic nerves and a retrocerebellar arachnoid cyst. The patient was treated with chemotherapy according to the CWS-Guidance 2014 and underwent hysterectomy. Now, 3 years after the end of therapy, she has been in ongoing remission. Family history was unremarkable for tumors or phenotypic abnormalities.

## Methods and results

Conventional karyotyping as well as chromosomal whole-genome microarray from peripheral blood lymphocytes revealed normal results.

Whole exome sequencing (WES) in patients 1, 2 and 4 was carried out using a probe-based capture method to enrich the target regions (IDT Coraville, IA, USA). Alignment to the reference genome (hg19 or hg38), variant calling and analysis was performed using an in-house pipeline based on SeqMule and Kggseq. In patient 3, WES was performed on the proband and her mother as previously reported with modification in capture kit, Roche NimbleGen’s SeqCap EZ Human Exome Library, v3.0 with 64 Mb of exonic sequence targeted (Roche NimbleGen, Inc., Madison, WI) and sequencer NovaSeq 6000 (Illumina, San Diego, CA). Variants were called using three callers, HaplotypeCaller (version 3.8-1-0-gf15c1c3ef), UnifiedGenotyper (version 3.8-1-0-gf15c1c3ef) and FreeBayes (version v0.9.14-24-gc292036). Variants passed the GATK hard filter (QD < 2.0, FS > 60.0, MQ < 40.0, MQRankSum < − 12.5, ReadPosRankSum < − 8.0, SOR > 3.0 for SNV and QD < 2.0, FS > 200.0, ReadPosRankSum < − 20.0, SOR > 10.0 for INDEL), ABHet is between 0.2 and 0.8, and called by at least two of three callers.

In case 1, all affected family members carried a heterozygous pathogenic *DICER1* variant c.2307C>G; p.(Tyr769*) (ENST00000393063). However, no further obvious pathogenic variants were detected by whole-exome sequencing (WES).

**Fig. 2:**
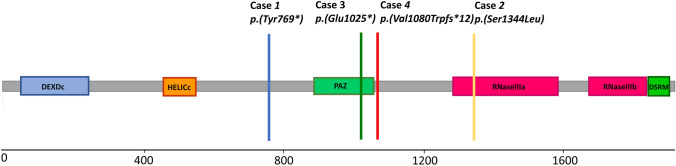
Diagram of *DICER1* variants in the four patients described here

In case 2, the heterozygous *DICER1* missense variant c.4031C>T; p.(Ser1344Leu) was identified in DNA of blood lymphocytes. This variant is located in the RNase IIIa domain (Fig. 2) and has previously been reported as a somatic mutation in various cancers, including Wilms tumour and as somatic hotspot mutation in uterine cancer [5, 6]. No other obvious pathogenic variants were detected by whole-exome sequencing (WES). Unfortunately, genetic testing could not be performed on patient’s parents nor on her two healthy siblings.

In case 3, the heterozygous nonsense variant c.3073G>T; p.(Glu1025*) in *DICER1* was identified in DNA of blood lymphocytes. Exome sequencing the patient did not reveal any other homozygous, compound heterozygous, X-linked or dominant pathogenic or likely pathogenic sequence variants in a gene known to be associated with a human phenotype (by OMIM listing), other than the known pathogenic *DICER1* variant.

In case 4 molecular genetics revealed a heterozygous variant in *DICER1* (Exon 21; c.3234_3237dupTGGC; p.(Val1080Trpfs*12)). This frameshift variant has not been described previously. The *DICER1* variant was inherited from the 50-year-old father without a history of tumours. Interestingly, heterozygosity for a frameshift variant c.5915_5916del, p.(Cys1972Tyrfs*11) (Chr6(GRCh38):g.157206936_157206937del) in *ARID1B* was detected in addition. This variant has not yet been described in the genome variant databases (gnomAD, ClinVar) and is suspected to be pathogenic. The variant was not detected in blood from the parents, suggesting a de novo origin. *ARID1B* mutations are associated with autosomal dominant inherited Coffin-Siris syndrome type 1 (OMIM 135900), which may in part explain some of the additional clinical features of the patient.

## Discussion

Most germline *DICER1* pathogenic variants are loss-of-function variants (LOF) [1]. LOF variants as in our patients 1, 3 and 4 are not likely to cause skewed miRNA processing, however, one cannot exclude the possibility that developmental defects might arise from *DICER 1* haploinsufficiency. Developmental delay and a syndromic phenotype combined with classical *DICER1*-associated tumours have been rarely reported and are described in association with a 14q32 deletion encompassing the *DICER1* locus [7, 8] (Table 1). One female patient with 14q32 deletion showed developmental delay, particularly in the field of speech in combination with mild facial dysmorphism (thick eyebrows, wide nasal base and bulbous nose) [9] as our patient 4 with a frameshift variant in *DICER1* and in *ARID1B*. This similarity of both patients raises the possibility that developmental delay and facial dysmorphism in both patients may be associated with *DICER1*. However, the situation is complicated by the fact that the patient also has the characteristics of Coffin-Siris syndrome and most additional phenotypic features like tracheomalacia and arachnoidal cyst may be well explained by the latter diagnosis [10]. Our patient 4 shows that, nevertheless, it cannot be excluded that in individual cases two rare conditions may occur simultaneously and that a comprehensive molecular genetic diagnosis is necessary in the presence of additional symptomatology.

**Table 1:** Patients with *DICER1* variants and syndromic features described in the literature

Phenotype	*DICER1* mutation or deletions including *DICER1*
6-year-old male, developmental delay, hypotonia, macrocephaly, obesity, and behavioral problems [8]	1,4 MB deletion 14q32
Mother: bilateral multinodular goiter and papillary thyroid carcinoma [8]	
15-year-old female, autism, coarse facial features, Sertoli-Leydig cell tumour, and Wilms’ tumour [8]	5 MB deletion 14q32
4-year-old male, developmental delay, congenital dysmorphic features, cystic nephroma, ciliary body medulloepithelioma, cerebral sarcoma, lung cyst, bifid uvula [9]	5,82 MB deletion 14q32
Male patient with Pierre-Robin sequence [15]	*DICER1*-mutation: p.(Tyr1511*)

Developmental delay is also a feature of GLOW syndrome (mosaic *DICER1* variant in the RNase IIIb domain) [2]. There are several similarities between the phenotypes of patient 2 and the two previously published patients with GLOW syndrome such as macrocephaly and macrosomia at birth, Wilms tumour, hydrocephalus, hypertelorism, lung cysts and developmental delay [2]. Unfortunately, segregation analysis in the family of patient 2 could not be undertaken and thus mosaicism in our patient cannot be ruled out, but allele distribution argues for a germline heterozygous *DICER1* variant. The detected missense variant c.4031C>T; p.(Ser1344Leu) within the RNase IIIa domain has been described so far only as somatic mutation in patients with Wilms tumour and other cancers, but not as germline variant, leaving a rest of uncertainty on the pathogenicity of this variant [11]. Interestingly, mutations within the RNase IIIa domain have been shown to phenocopy mutations in the RNase IIIb domain presumably due to the constrained proximity of the RNase IIIa and RNase IIIb as shown by structural and evolutionary coupling analyses [6]. This constrained proximity could also explain why the presumed germline variant in patient 2 leads to a phenotype similar to the one described in the two patients with GLOW syndrome associated with mosaic DICER1 mutation in the RNase IIIb domain, suggesting a genotype–phenotype relationship with missense mutations in RNase III. Whether a missense variant as in patient 2 is sufficient to lead to tumor development or a second somatic hit in *DICER1* is needed, as presumed in patients with LOF variants, remains speculative.

Pierre-Robin sequence and other skeletal abnormalities as described in patient 1 may also be a rare phenotypic presentation of a *DICER1* variant. Pierre-Robin sequence is a craniofacial anomaly which includes mandibular hypoplasia, glossoptosis and often cleft palate. There are several genetic causes leading to this phenotype [12]. Interestingly, DICER has been shown to play a role in nucleolar function, and heterozygous pathogenic variants in genes involved in nucleolar homeostasis have been identified to cause various craniofacial disorders [13, 14]. One case of Pierre-Robin sequence associated with *DICER1* variant was previously described [15]. In mice, a conditional *Dicer1* deletion leads to late embryonic lethality and severe craniofacial dysmorphism, including a secondary cleft palate [16].

Abnormalities in optic nerves as in patient 4 were previously described in patients with *DICER1* germline variants [17]. In a mouse model, conditional *Dicer1* deletion in the retina led to developmental disorder of retinal cells [18]. Although ocular involvement has to be discussed in this patient in context of the identified *ARID1B* variant, in Coffin-Siris syndrome ocular findings are more likely to manifest as strabismus, nystagmus, cataract, hypophoria, astigmatism, hypermetropia and anisomyopia [19], with optic nerve hypoplasia being described only occasionally [20].

In summary, germline pathogenic *DICER1* variants may not only be associated with the occurrence of certain tumour types, but might also rarely include developmental features, like Pierre-Robin sequence, developmental delay, facial dysmorphisms, and ocular abnormalities. Actually, it is rather surprising that *DICER1* pathogenic germline variants do not lead to an even more severe clinical phenotype, since DICER1 is an absolutely central molecule in RNA interference: Dicer catalyses the first step of RNA interference and initiates the formation of the RNA-induced silencing complex (RISC), where argonaute endonuclease, is able to degrade mRNA whose sequence is complementary to the resulting siRNA. Due to this fundamental mechanism, effects in all kinds of cells are conceivable. Nevertheless, we acknowledge that the role of concomitant pathogenic variants in other genes cannot be ruled out, or may indeed modify the phenotype such as in patient 4. The coincidence of developmental phenotypes and pathogeneic *DICER1* variant merits further evaluation.

## Data Availability

The raw data supporting the conclusions of this article will be made available by the authors, without undue reservation.
